# EEG Cross-Subject Taste Classification Method: A Meta-Learning Wavelet Graph Convolutional Neural Network Under Sweet and Bitter Stimuli

**DOI:** 10.3390/bios16050295

**Published:** 2026-05-19

**Authors:** He Wang, Hong Men, Yan Shi

**Affiliations:** 1 School of Automation Engineering, Northeast Electric Power University, Jilin 132012, China; 1202300098@neepu.edu.cn (H.W.); shiyan@neepu.edu.cn (Y.S.); 2 School of Transportation, Jilin Tiedao University, Jilin 132299, China

**Keywords:** EEG detection, cross-subject, taste classification, meta-learning, wavelet graph convolutional neural network

## Abstract

Traditional taste evaluation relies heavily on manual sensory analysis, which is highly subjective and inefficient with poor cross-individual generalization, limiting its application in industrial flavor detection. To achieve accurate cross-subject taste recognition, this paper proposes an electroencephalogram (EEG) classification method based on a meta-learning wavelet graph convolutional neural network (ML-WGCNet) under sweet- and bitter-taste stimuli. Sucrose (sweetness) and quinine (bitterness) were used as stimulation sources, each prepared at six concentration gradients, including a water control. EEG signals were detected from 20 subjects. First, the Morlet wavelet transform was applied to decompose the EEG signals in the time–frequency domain, extracting the maximum and average energy values from five frequency bands as core features. A graph structure was then constructed using electrodes as nodes and Pearson correlation coefficients between electrodes as edge weights. A lightweight graph convolutional neural network (GCN) is employed to model spatial correlations among brain regions. Finally, by integrating a meta-learning framework and adopting leave-one-subject-out cross-validation, the model can rapidly adapt to new subjects. The experimental results show that the proposed method achieves average accuracies of 76.03% and 77.01% in cross-subject classification of sweet and bitter tastes, respectively. The corresponding precision values are 79.94% and 79.53%, the recall values are 75.77% and 78.51%, and the F1-scores are 78.24% and 78.08%, respectively, demonstrating that the proposed model significantly outperforms existing mainstream EEG classification methods.

## 1. Introduction

With consumers’ increasingly refined demands for flavor quality, traditional reliance on manual sensory evaluation presents limitations such as strong subjectivity, significant individual variation, and low evaluation efficiency [1]. These shortcomings make it difficult to meet the requirements for large-scale production and precise quality control in industry [1]. Against the backdrop of interdisciplinary integration between sensory science and neural engineering, research on electroencephalogram (EEG) taste recognition has gradually emerged [2]. EEG signals directly reflect the genuine responses of the brain’s neural activity, enabling an objective and unbiased evaluation of the brain’s neural representation and perceptual processing of external stimuli, and have been widely applied in medical diagnosis, emotion recognition and other fields [3,4]. As an objective physiological indicator reflecting the brain’s neural activity, EEG signals can capture the real-time response characteristics of the cerebral cortex under taste stimulation, providing direct evidence for deciphering the neural perception mechanisms of taste [5,6]. Sweetness and bitterness, as fundamental tastes with a strong antagonistic relationship, represent core dimensions influencing flavor acceptance and are thus well-suited for validating and constructing EEG recognition models [7,8]. EEG recognition of sweet and bitter tastes contributes to establishing an objective evaluation system for taste perception, overcoming the bottlenecks of manual evaluation, and facilitating precise and standardized flavor formulation in product research and development [9]; it also offers a new technical approach for rapid quality and safety inspection, promoting the advancement of the industry toward intelligence and personalization.

However, the practical application of EEG taste recognition still faces numerous challenges in EEG data processing. EEG signals inherently possess low signal-to-noise ratios and are susceptible to interference from artifacts such as ocular and muscular activity, necessitating complex steps such as filtering, noise reduction, and feature extraction to isolate and characterize valid signals [10,11]. Furthermore, neural responses exhibit highly nonlinear characteristics in the time and frequency domains [12]. How to compute discriminative features with high specificity and achieve high-performance recognition remains a key issue in the field of EEG recognition [13,14]. To address the needs for precision, standardization, and personalization in flavor formulation within product research and development, an even more prominent challenge lies in achieving cross-subject recognition [2]. Due to innate differences in cortical structure and taste sensitivity among individuals, coupled with factors such as dietary preferences and physiological states that further contribute to the individual specificity of EEG response patterns, recognition models trained on data from a single subject or a small sample often experience a significant drop in accuracy when applied to new subjects. Establishing effective cross-subject EEG recognition methods, mitigating the impact of individual differences, and ensuring model generalizability and adaptive ability are core problems that must be solved for EEG taste recognition technology to transition from laboratory research to industrial application [15]. With the advancement of artificial intelligence, deep learning algorithms offer significant advantages in feature computation [16], learning optimization [17], and adaptive classification [18]. These strengths provide effective technical approaches for in-depth research on cross-subject EEG analysis.

In research on cross-subject EEG studies, many scholars have made significant contributions. The optimized deep learning framework, for example, enhances EEG-based cross-subject recognition, making it effective in capturing brain activity patterns [19,20]. Zhou et al. proposed an EEG deep domain adaptation (DDA) method; by combining feature distribution alignment with adversarial learning, this approach effectively mitigates inter-subject variability [21]. Morvay et al. introduced an optimal transport-based Wasserstein barycenter transfer (WBT) method, which employs unsupervised domain adaptation to align EEG feature distributions across different subjects [22]. Zhang et al. developed an evolutionary programming ensemble learning (EPEL) framework by automatically evolving and integrating multi-structure neural networks to address inter-subject EEG variability [23]. Additionally, Liu et al. proposed a spatio-temporal hybrid network based on domain adaptation and dynamic graph attention (ST-DADGAT), which models the spatial topological correlations of EEG channels [24]. Huang et al. proposed a novel multiple regularized knowledge transfer (MRKT) learning method, which was built on the maximum mean discrepancy minimization framework with Riemannian tangent space features [25]. Peng et al. introduced an EEG feature transfer and semi-supervised learning method by simultaneously conducting distribution alignment and estimating target subject emotion labels in a shared subspace [26]. Li et al. proposed a cross-domain spiking convolutional network (CDSCN), which applied the spiking convolutional block [27]. Although these methods have advanced the development of cross-subject EEG recognition technology, notable limitations remain. First, most methods focus only on general EEG scenarios, without fully exploiting the synergistic value of time–frequency rhythm features and spatial brain region correlations, resulting in limited feature discriminability. Second, existing methods lack tailored feature extraction schemes designed for the non-stationary nature of EEG signals, making it difficult to match the specific neural response patterns of taste-evoked EEG, and leading to insufficient generalization ability and classification accuracy in cross-subject recognition scenarios.

Meta-learning, as a machine learning paradigm focused on “learning to learn”, enables rapid adaptation to unknown distributions of new tasks by extracting generalizable knowledge from multiple tasks [28]. This provides a core solution for addressing the generalization challenge in cross-subject EEG recognition caused by individual differences. Awan et al. ensembled a meta-classifier for cross-subject emotion recognition (MC) [29]. Chen et al. constructed symmetric positive definite matrices and meta-transfer learning (MTL); this model characterizes the spatial information of EEG signals via symmetric positive definite matrices [30]. Li et al. combined multi-scale residual networks and meta-transfer learning (MSRN-MTL), effectively mitigating generalization issues arising from individual differences [31]. Wang et al. introduced a Meta-learning and Anes-MetaNet; the model employs a convolutional neural network to handle inter-subject variability and extracts temporal features via long short-term memory [32]. Although meta-learning provides an effective approach for cross-subject adaptation, existing meta-learning EEG classification methods largely focus on optimizing model parameter initialization or employing simple ensemble strategies; they have not yet developed an integrated framework tailored to taste-related EEG that combines “time–frequency features, spatial modeling, and meta-adaptation”. Moreover, most methods do not fully leverage the concentration-dependent patterns of brain region activation under taste stimulation, limiting the accuracy and robustness of cross-subject classification. Building upon the fast-adaptation advantages of meta-learning, this paper further optimizes feature extraction and model architecture to construct a cross-subject recognition system specifically adapted for taste-related EEG.

In summary, to address the challenges of large individual differences, insufficient feature discriminability, and poor model generalization in cross-individual taste EEG recognition, this paper proposes an adaptive deep learning method that enables high-precision cross-subject EEG classification of sweet and bitter taste concentrations, providing technical support for objective taste evaluation and industrial applications. The main contributions of this paper are as follows:

(1) Through the analysis of EEG signals under the stimulation of different sweet and bitter concentrations, distinct brain region activation patterns between the two taste types are clarified. For sweet tastes, activation progressively expands from primary sensory regions to a distributed cortical network encompassing sensory and cognitive areas as the concentration increases. For bitter taste, activation exhibits alertness-related activation at low concentrations and focused activation in core regions at high concentrations. These findings provide reliable physiological mechanistic support for the precise discrimination of taste concentrations.

(2) Wavelet transform (WT) and a graph convolutional neural network (GCNet) are innovatively integrated. Wavelet decomposition captures multi-band neural rhythmic features, while the combination of maximum and average energy quantifies the non-stationary characteristics of the signal. GCNet is then employed to mine spatial correlations among electrodes, forming a synergistic strategy of “time–frequency feature extraction, energy representation, and spatial modeling”. This approach significantly enhances the discriminability of taste-related EEG signals.

(3) A meta-learning cross-subject training framework is constructed. Data from individual subjects are treated as independent meta-tasks, and a leave-one-subject-out cross-validation strategy combined with a rapid adaptation mechanism is adopted to achieve efficient adaptation of the model to new subjects. Experiments confirm that this method achieves favorable classification performance in cross-subject classification of sweet and bitter tastes, significantly outperforming mainstream methods, thereby laying a foundation for the industrial application of objective taste evaluation technology.

The main structure of this paper is as follows: Section 1 introduces the research background, existing challenges, related progress, and the core contributions of this study on cross-subject recognition of taste-related EEG. Section 2 presents the materials and methods, including the preparation of taste stimulation samples, the EEG acquisition system, the experimental protocol for taste detection, the construction of the wavelet graph convolutional network (WGCNet), and the meta-learning-based cross-subject training process. Section 3 presents and discusses the results, showing the spatial response patterns of brain regions under sweet and bitter taste stimulation, the ablation experiment results of WGCNet, and the comparison and analysis of cross-subject classification performance, as well as research limitations and future plans. Section 4 presents our conclusions, summarizing the findings and research value of this study.

## 2. Materials and Methods

### 2.1. Samples and EEG Acquisition System

In this experiment, sucrose and quinine serve as standard reference substances for preparing sweet- and bitter-tasting solutions, respectively. For the preparation of sweet-tasting solutions at different concentrations, 0.3 g, 0.6 g, 1.2 g, 2.4 g, and 2.8 g of sucrose were weighed and dissolved separately in 100 mL of distilled water to obtain five distinct concentrations. For the preparation of bitter-tasting solutions at different concentrations, 0.0002 g, 0.0004 g, 0.0008 g, 0.0016 g, and 0.0032 g of quinine were weighed and dissolved separately in 100 mL of distilled water to obtain five distinct concentrations.

**Figure 1:**
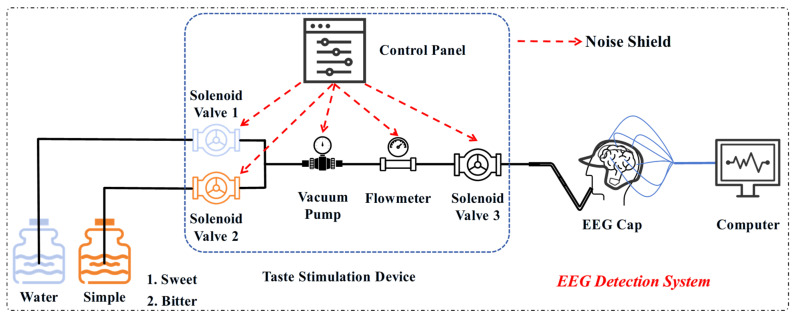
Structure of the EEG detection system.

The EEG acquisition system used was the NCERP-P system, manufactured by Shanghai Nuocheng Electric Co., Ltd. EEG data from 21 channels related to taste were collected at a sampling rate of 256 Hz. The structure of the detection system is shown in Figure 1. According to the International 10–20 electrode placement system, electrodes were precisely positioned at the following locations on the EEG cap produced by Wuhan Green Tech Co., Ltd.: Fz, Cz, Pz, T3, T4, C3, C4, Fp1, Fp2, F7, F8, T5, T6, O1, O2, F3, F4, P3, P4, A1, and A2. The reference electrode was placed on the bilateral earlobes. Conductive gel was applied at each electrode, and the contact conditions were adjusted to ensure that the impedance of each channel remained below 10 kΩ. Upon completion of system assembly, baseline signal acquisition and equipment calibration were implemented sequentially to guarantee the acquisition of high-fidelity EEG signals with minimal artifacts. In order to achieve accurate regulation and high-sensitivity detection of taste-evoked EEG responses, a custom-developed liquid delivery module was seamlessly integrated with the EEG recording system. The core components of this liquid delivery module include a STM32F103ZE main control board (supplied by Yehuo Technology Co., Ltd., Dongguan, China), a S15S-53J miniature vacuum pump (manufactured by Hailin Technology Co., Ltd., Chengdu, China), an sfos-1037v-01 electromagnetic valve (provided by Yehuo Electronic Technology Co., Ltd., Dongguan, China), and an AB32-S21P020C-11R liquid flow sensor (produced by ODE Limited, Hong Kong, China). Specifically, the main control board serves as the central command unit, which not only allows for precise tuning of liquid flow velocity and dispensed volume but also executes real-time and accurate control over the on-off status of the electromagnetic valve and the operational parameters of the vacuum pump. The external part of the device is equipped with a soundproof cover and sound-absorbing foam to effectively reduce operational noise, thereby creating a quiet and stable experimental environment. Consequently, a dedicated taste stimulation device (Figure 1) was also designed and applied in this study, ensuring that liquid stimuli entered the subject’s mouth at a constant flow rate and volume. This minimized human error and guaranteed accuracy and reproducibility throughout the stimulation process.

### 2.2. Taste Detection Experiment

In this study, we designed and conducted a taste-related EEG stimulation experiment. A total of 20 healthy adult subjects were recruited for the experiment, including 10 males and 10 females, aged 20–24 years, with a mean age of 22.15 ± 1.23 years, and with no history of smoking or right-handedness. Pure water stimulation was included as a control condition to rule out the influence of non-taste factors on EEG activity. To ensure the balance of the data, the sample size for each subject, each concentration of sweet/bitter taste, and the control group of pure water was exactly the same. All samples were processed according to the same pre-treatment, segmentation, and artifact removal procedures, with no sample bias.

To ensure the stability and reliability of the EEG data collected during the taste experiment, the following experimental protocols were implemented:

(1) Experimental Environment Control: EEG experiments are highly sensitive to external interference. To minimize the effects of environmental noise and lighting variations, the experiment was conducted in a quiet, isolated laboratory equipped with acoustic isolation and electromagnetic shielding systems to avoid external disturbances. Adjustable lighting and light-blocking devices were installed indoors to maintain a soft and stable visual environment. The laboratory was cleaned and ventilated prior to the experiment, in order to prevent odors from affecting the participants’ mood. During the experiment, room temperature was maintained at 22 ± 2 °C and relative humidity at 50 ± 10% RH, in order to ensure participant comfort and EEG signal stability.

(2) Participant State Requirements: Participants were instructed to ensure adequate sleep the night before the experiment, so that they could participate in a good mental state. On the day of the experiment, consumption of caffeine, spicy foods, or any substances that may affect neural activity was prohibited. The use of perfumes or other odor-stimulating products was also avoided, so as to prevent cross-influences between taste and olfaction.

(3) Physiological Preparation and Taste Control: To avoid signal distortion caused by high scalp resistance, the participants were required to wash their hair on the day of the experiment and refrain from using oily hair care products. The experiment was generally scheduled approximately two hours after a meal, in order to minimize the effects of hunger or satiety on taste sensitivity. Before the experiment, the participants rinsed their mouths with water to remove residual taste substances, ensuring the independence and comparability of each stimulus.

(4) Equipment and Electromagnetic Protection: Only the EEG acquisition system, stimulus-control computer, and essential auxiliary devices remained in the laboratory, eliminating interference from other electromagnetic emission sources. Grounding checks and signal calibration were performed before the experiment, in order to ensure that the impedance of all electrodes was below 10 kΩ. Baseline signal detection was completed before the formal experiment began, in order to guarantee stable system operation and the acquisition of authentic and effective signals.

The specific steps of the formal experiment were as follows:

(1) Preparation Phase.

After entering the laboratory, the participant first underwent identity verification and received an explanation of the experiment. The experimenter provided a detailed introduction to the experimental purpose, task requirements, and precautions, and informed consent was obtained. Subsequently, the participant was guided to a comfortable experimental chair, maintaining an upright sitting posture with a relaxed body. Both hands were placed naturally on the table surface, and movement was minimized to reduce the impact of motion artifacts on EEG signals.

(2) Electrode Placement and Calibration.

Following the International 10–20 electrode placement system, electrodes were precisely positioned at the following locations on the EEG cap manufactured by Wuhan Green Tech Co., Ltd. (Wuhan, China): Fz, Cz, Pz, T3, T4, C3, C4, Fp1, Fp2, F7, F8, T5, T6, O1, O2, F3, F4, P3, P4, A1, and A2. The reference electrode was placed on both earlobes. Conductive gel was applied at each electrode, and the contact conditions were adjusted to ensure that the impedance of each channel remained below 10 kΩ. After installation, baseline signal detection and device calibration were performed to ensure stable EEG signals without significant artifacts.

(3) Taste Stimulus Presentation.

This experiment adopted a randomized presentation design. Six concentration gradients (including water) were set for both sweet and bitter solutions, and one concentration was randomly selected for stimulation each time. Six distinct gradients of taste stimulation solutions were freshly prepared and assigned the labels S0-S5 and B0-B5 two hours prior to the formal initiation of the experiment, with ultrapure distilled water (designated as concentration 0) employed as the blank control.

**Figure 2:**
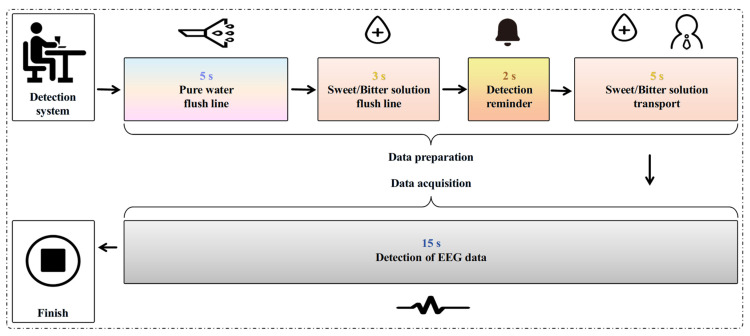
Timing sequence for each experiment.

**Figure 3:**
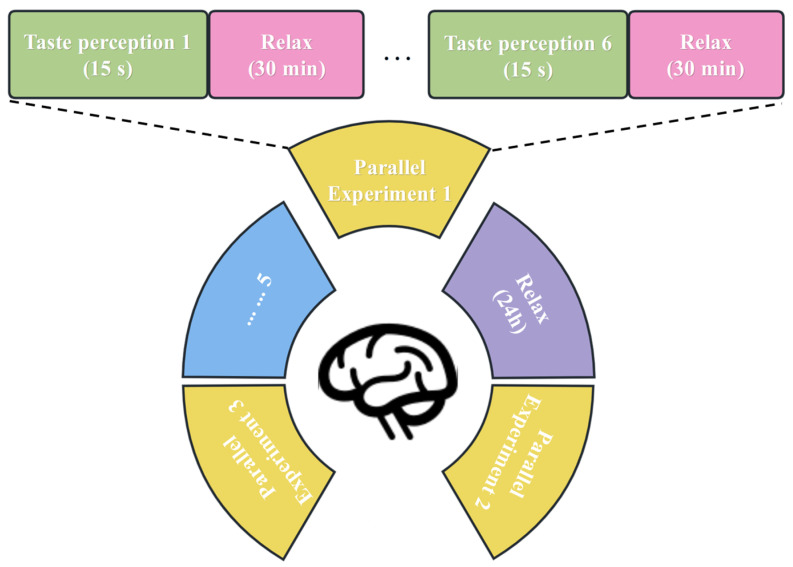
Parallel experimental process.

All formulated solutions were then placed in a 37 °C constant-temperature incubator for storage, so as to maintain a stable temperature consistent with that of the human oral cavity. The detailed implementation procedures were as follows:

(1) Prior to the initiation of each experimental trial, the start button was activated to trigger the distilled water rinsing mode of the taste stimulation system. Within approximately 5 s, the system rapidly delivered distilled water to the nozzle, enabling thorough flushing and cleansing of the internal pipelines. This pre-trial rinsing step was implemented to eliminate residual substances that might otherwise introduce interference to the experimental outcomes. (2) After the distilled water rinsing process, the system automatically shifted to the solution rinsing mode. The experimental solution was pumped to the nozzle at an elevated flow rate for 3 s, ensuring the complete expulsion of any residual distilled water within the pipelines and the full priming of the pipelines with the experimental solution. At the conclusion of the rinsing phase, a buzzer emitted a 0.5 s alert to notify the participant of the imminent start of the trial. (3) Two seconds after the termination of the rinsing procedure, the taste stimulation system transitioned to the solution stimulation mode. A 2.5 mL aliquot of the stimulation solution was dispensed at a flow rate of 0.5 mL/s over a 5 s duration, with uniform delivery targeted at the region 0.25–0.5 cm above the center of the participant’s tongue. Participants were instructed to retain the solution in their oral cavity without swallowing for 15 s, facilitating the adequate induction of taste-evoked EEG signals. (4) Upon the completion of EEG data acquisition, the buzzer was activated again to signal the end of the experimental stimulation phase. The participants then performed three rounds of oral rinsing, each with 50 mL of purified water, in order to thoroughly eliminate any residual solution from the oral cavity, thereby preventing taste carryover and cross-stimulation interference. To mitigate taste fatigue, the participants were given a 30 min rest interval before commencing the subsequent experimental trial. (5) Each participant received only one type of taste stimulus per session, with a total of 5 sessions. In each session, six concentration gradients were randomly selected, and each concentration was repeated for 6 rounds, with the presentation order randomized to reduce order effects. EEG data for pure water were also collected as a control in each session. After each round, the participant rested for 24 h. The entire experiment was monitored by researchers, and the trial could be terminated at any time if discomfort occurred. The timing sequence for each experimental trial is shown in Figure 2, and the overall parallel experimental process is illustrated in Figure 3. (6) The sampling frequency of EEG detection is 256 Hz, which can effectively capture the dynamic responses of taste perception. Each taste concentration stimulation generated six parallel sets of 15 s EEG data. During the sample segmentation process, each sample lasted for 1 s, meaning that each subject would have 90 samples under each taste concentration stimulation.

Before performing deep feature extraction and classification of EEG signals, the raw taste-related EEG data needed to be preprocessed. First, a 0.5–45 Hz bandpass filter was applied; its core purpose was to lock onto the effective rhythm frequency bands of taste-evoked EEG activity, fully retaining the five key frequency bands—Delta (0.5–4 Hz), Theta (4–8 Hz), Alpha (8–13 Hz), Beta (13–30 Hz), and Gamma (30–45 Hz)—while filtering out very low-frequency baseline drift and ineffective high-frequency noise above 45 Hz [2]. In practical engineering implementation, digital bandpass filters have non-ideal transition band characteristics. The cutoff at 45 Hz is not abrupt, and some residual high-frequency components remain in the 45–50 Hz range [9,33]. Therefore, we added a 47 Hz lowpass filter as a protective measure, further suppressing the residual energy in the 45–50 Hz range, preventing edge components of power-line interference from entering the effective frequency band, and reducing the interference burden for subsequent precise notch filtering. Furthermore, the 50 Hz notch filter is a narrowband interference suppression technique, which is functionally complementary to the bandpass and lowpass filters [5]. In laboratory environments, 50 Hz power-line interference has strong energy and sharp peaks, which can easily pass through the transition band of a conventional bandpass filter. The bandpass filter only provides broadband attenuation and cannot precisely eliminate this strong, single-frequency interference. The 50 Hz notch filter can deeply suppress power-line noise at a specific frequency, while maximally preserving the effective rhythm signals within 0.5–45 Hz, avoiding the loss of useful information. In summary, the preprocessing in this study strictly followed the order of broadband filtering first, followed by precise noise suppression. Specifically, we first applied a 0.5–45 Hz bandpass filter to define the effective signal range; then, we used a 47 Hz lowpass filter to compensate for the bandpass transition band defect; finally, we applied a 50 Hz notch filter to remove residual interference. This order prevented the progressive propagation of interference and minimized the phase shift and amplitude distortion caused by cascaded filtering.

### 2.3. Wavelet Graph Convolutional Neural Network

**Figure 4:**
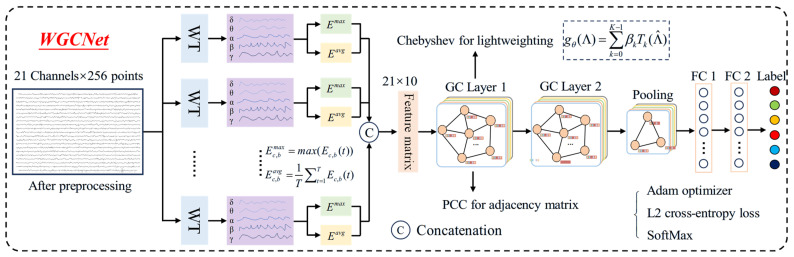
The main structure of WGCNet.

EEG data are characterized by two essential properties: multiscale rhythmicity and spatial nonstationarity [2,5,6]. Taste-evoked EEG is a strongly non-stationary signal; its neural responses have instantaneous peaks and sustained activation over time. Classical bandpass filtering would lose this key dynamic response information. In contrast, WT can localize signals in both the time and frequency domains; it not only extracts standard frequency bands, but also accurately captures energy changes at each time point, making it more suitable for transient, evoked neural activities such as taste perception. First, WT is applied to the preprocessed EEG signals for time–frequency decomposition [27]. This method adaptively captures transient rhythmic events at each electrode, yielding five frequency bands: Delta, Theta, Alpha, Beta, and Gamma. Stable and reproducible correlations exist between rhythms of different frequencies and specific physiological states or cognitive functions of the brain [34]. These correlations provide a biophysically grounded and functionally well-defined standardized analytical framework for understanding and decoding brain states. Subsequently, to address the nonstationary nature of the signals, the maximum and average energy within each frequency band are computed via WT, representing the salient nonstationary features and overall trend characteristics of each band [27]. Wavelet energy calculation is stable and highly noise-resistant. Taste-evoked EEG signals are strongly non-stationary, and energy can directly reflect both the intensity and dynamic changes in neural activity. Maximum energy captures the peak of neural responses, corresponding to the moment of strongest activation elicited by taste stimuli. Average energy represents the overall level of neural activity across the entire time window, reflecting the sustained characteristics of taste processing. The two measures are complementary, enabling comprehensive quantification of neural rhythm differences across the Delta, Theta, Alpha, Beta, and Gamma frequency bands, thereby enhancing the ability to discriminate between sweet and bitter tastes as well as their concentrations. Next, graph structures are constructed from the features computed for different electrodes, where electrodes serve as graph nodes and the Pearson correlation coefficients between electrodes form the adjacency matrix. The calculation of Pearson correlation coefficients is efficient, stable, and suitable for quickly constructing graph structures for low-channel EEG; it can quantify the functional connection strength between electrodes. Finally, a WGCNet is designed to classify EEG data corresponding to different concentrations of sweet and bitter tastes. Figure 4 shows the main structure of WGCNet, and the model consists of the following four main modules:

(1) Time–Frequency Decomposition via WT.

The rhythmic characteristics of EEG signals are distributed across different frequency ranges, and specific frequency bands are closely associated with the neural processing of taste perception. To accurately capture the transient rhythmic responses of each electrode under taste stimulation, this study employs the Morlet wavelet as the mother wavelet for time–frequency decomposition [35,36]. The Morlet wavelet offers both excellent temporal localization and frequency resolution, enabling adaptive tracking of instantaneous frequency changes in EEG signals; its mathematical expression is as follows:

(1) $$\varphi (t)=\pi^{-1/4}\left(e^{jw_{0}t}-e^{-w_{0}^{2}/2}\right)e^{-t^{2}/2}$$

where $w_{0}=5$ represents the carrier frequency, which ensures a balanced resolution of the wavelet in both the time and frequency domains, and φ(t) is the decomposition result. During the pre-experiment, we also examined the influence of different wavelet basis functions on model classification performance. The results of this discussion were presented in Table S1 of the Supplementary File. The specific procedure for time–frequency decomposition is as follows:

Step 1: After preprocessing the raw EEG signals to remove baseline drift and power-line interference, the Morlet wavelet is applied to perform continuous WT on the EEG signals from 21 channels (each channel comprising 256 sampling points). This step calculates the inner product between the signal and wavelet bases at different scales, yielding a time–frequency coefficient matrix with dimensions *T* × *F*, where *T* = 256 represents the number of timepoints and *F* corresponds to the number of frequency bands defined by the scaling.

Step 2: Based on the physiological rhythmic characteristics of EEG signals, the time–frequency coefficients are divided into five typical frequency bands: Delta, Theta, Alpha, Beta, and Gamma. Each band corresponds to an independent sub-matrix of time–frequency coefficients, enabling the separate extraction of distinct neural rhythmic features.

Through wavelet time–frequency decomposition, the one-dimensional (256 × 1) time-domain EEG signal is transformed into a two-dimensional (256 × 5) time–frequency feature matrix. This representation retains the temporal dynamics of the signal while clarifying its frequency–rhythm distribution, thereby providing a structured data foundation for subsequent feature quantification.

(2) Computation of Maximum and Mean Wavelet Energy Values.

EEG signals evoked by taste stimulation exhibit marked non-stationary characteristics. Variations in energy across different frequency bands directly reflect the intensity and dynamic trends of neural activity. To quantify both the non-stationary features and the overall patterns within each frequency band, this study calculates the maximum and mean energy values from the time–frequency coefficient matrix of each band as core features.

Wavelet Energy Calculation: For the *c*-th channel and the *b*-th frequency band, let $W_{c,b}\in {ℜ}^{T\times F_{b}}$ denote the time–frequency coefficient matrix of the band (where $F_{b}$ represents the number of frequency points in that band). The instantaneous energy at each time point *t* is defined as follows:

(2) $$E_{c,b}(t)=\sum_{f=1}^{F_{b}}{\left|W_{c,b}(t,f)\right|}^{2}$$

where $\left|W_{c,b}(t,f)\right|$ represents the magnitude of the time–frequency coefficient. The energy calculation reflects the intensity of neural activity within that frequency band at the corresponding timepoint.

Extraction of maximum energy values: For each channel and each frequency band, the maximum value is extracted from the instantaneous energy sequence $E_{c,b}(t)$(t=1,2,⋯,T). This metric highlights the peak neural responses elicited by taste stimulation, captures the most prominent rhythmic activity characteristics, and reflects the intensity of key activation moments during neural processing. The calculation is expressed as follows:

(3) $$E_{c,b}^{max}=max(E_{c,b}(t))$$

Extraction of Mean Energy Values: The mean value of the instantaneous energy sequence is calculated for each channel and each frequency band. This metric characterizes the average level of neural activity within the band over the entire stimulus period, reflecting the overall activation trend of neural rhythms during taste perception. The calculation is expressed as follows:

(4) $$E_{c,b}^{avg}=\frac{1}{T}\sum_{t=1}^{T}E_{c,b}(t)$$

Ultimately, the feature vector for each channel has a dimension of 2 × 5 = 10 (5 frequency bands × 2 energy metrics). Across all 21 channels, this yields an original feature response matrix of size 21 × 10, enabling cross-subject classification based on the time–frequency characteristics of the EEG signals.

(3) Graph Structure Construction for EEG Features.

Advanced graph-based EEG classification models can significantly improve feature representation and classification ability. EEG electrodes can be naturally modeled as graph nodes, and the functional connections between electrodes as graph edges. Graph computing can precisely mine spatial topology and functional synergy among brain regions, and it also adapts well to the non-Euclidean structure and strong spatial dependencies of EEG; therefore, applying it to EEG recognition has strong physiological and technical justification [37]. In the graph modeling of this paper, the nodes are the 21 EEG electrodes. The edge set consists of the connections between all electrode pairs. The adjacency matrix is a connection strength matrix, where each connection is quantified using the Pearson correlation coefficient. The edge set defines the structure of connections between electrodes, while the adjacency matrix provides the numerical expression of those connection strengths. The relationship between the two is that of “structural definition” versus “numerical representation”. The specific procedure is as follows:

Step 1: Definition of Graph Nodes: The 21 EEG electrodes are defined as graph nodes. The feature vector of each node corresponds to the 10-dimensional energy feature set derived from the corresponding electrode, forming a graph node feature matrix.

Step 2: Construction of the Adjacency Matrix: The Pearson Correlation Coefficients are adopted to quantify the strength of feature-based associations between electrodes, serving as the edge weight of the graph (adjacency matrix).

Step 3: Standardization of the Adjacency Matrix: Since the adjacency matrix used in graph convolution is undirected, the absolute values of its entries are taken.

By constructing this graph structure, the initially dispersed electrode features are integrated into spatially correlated, structured data, effectively exploring the cooperative processing of information among brain regions and providing a basis for capturing the distributed neural mechanisms underlying taste perception. The procedure for building the Graph Convolutional Network (GCN) is detailed below:

In a GCN, G={V,ε,A} denotes an undirected graph [38], where *V* represents the set of nodes, ε represents the set of edges, and *A* is the adjacency matrix that defines the connections between nodes. The structure of the graph can be expressed by the Laplacian matrix (*L*), which is formulated as follows:

*L* = *D* − *A* (5)

where *D* denotes the degree matrix, and the *i*-th diagonal element thereof is calculated as follows:

(6) $$D_{ii}=\sum_{j}A_{ij}$$

In an undirected graph, *L* is a positive semidefinite matrix, and $A_{ij}=A_{ji}$. It undergoes spectral decomposition to yield:

(7) $$L=U\Lambda U^{-1}=U\left[\begin{array}{ccc} \lambda_{1} & & \\ & \ddots & \\ & & \lambda_{n} \end{array}\right]U^{-1}$$

where $U={\left\{u_{i}\right\}}_{i=1}^{n}$ denotes mutually orthogonal eigenvectors, which serve as the Fourier basis. $\Lambda =diag({\left\{\lambda_{i}\right\}}_{i=1}^{n})$ represents a symmetric matrix, and $\lambda_{i}$ indicates the eigenvalues corresponding to *u_i_*. Given that U is an orthogonal matrix ($UU^{T}=E$), the above expression can be rewritten as follows:

(8) $$L=U\Lambda U^{-1}=U\Lambda U^{T}$$

The graph Fourier transform converts signals defined in the spatial domain to the spectral domain in order to facilitate convolution operations, followed by an inverse transformation back to the spatial domain. The conversion of a graph signal *x* from the spatial domain to the spectral domain can be formulated as follows:

(9) $$\hat{x}=F(x)=U^{T}x$$

The inverse graph Fourier transform of the signal is formulated as follows:

(10) $$x=F^{-1}(x)=U\hat{x}$$

Accordingly, graph convolution can be formulated as follows:

(11) $$\begin{array}{cc} x{*}_{G}g & =F^{-1}(F(x)⊙F(g))\\ & =U((U^{T}x)⊙(U^{T}g))\\ & =U\left[\left(\begin{array}{c} \hat{x}(\lambda_{1})\\ \vdots \\ \hat{x}(\lambda_{n}) \end{array})⊙(\begin{array}{c} \hat{g}(\lambda_{1})\\ \vdots \\ \hat{g}(\lambda_{n}) \end{array}\right)\right]\\ & =U\left[\begin{array}{ccc} \hat{g}(\lambda_{1}) & & \\ & \ddots & \\ & & \hat{g}(\lambda_{n}) \end{array}\right]\left[\begin{array}{c} \hat{x}(\lambda_{1})\\ \vdots \\ \hat{x}(\lambda_{n}) \end{array}\right]\\ & =U\left[\begin{array}{ccc} \hat{g}(\lambda_{1}) & & \\ & \ddots & \\ & & \hat{g}(\lambda_{n}) \end{array}\right]U^{T}x \end{array}$$

where ${*}_{G}$ represents graph convolution, ⊙ denotes the Hadamard product, and *g* is the convolution kernel. Let

(12) $$g_{\theta }(\Lambda )=diag(U^{T}g)$$

Ultimately, the expression of graph convolution can be converted into

(13) $$x{*}_{G}g_{\theta }=Ug_{\theta }(\Lambda )U^{T}x$$

The trainable parameters of the convolution kernel can be formulated as follows:

(14) $$g_{\theta }(\Lambda )=diag(\theta_{i})\begin{array}{cc} & \theta_{i} \end{array}\in {ℜ}^{n}$$

However, GCN involves computationally expensive eigendecomposition. Moreover, the number of graph convolution parameters equals the number of graph nodes. Consequently, an excessive number of convolution parameters can impair decision performance. It is noteworthy that the graph convolution kernel is inevitably multiplied by the matrix *U*, leading to a computational complexity of O(*n*^2^) for GCN. To address this issue, Chebyshev polynomials are adopted in this study to simultaneously reduce the number of parameters and lower the computational complexity. Initially, a polynomial approximation is applied to the convolution kernel, which is expressed as follows:

(15) $$g_{\theta }\left(\Lambda \right)=\sum_{k=0}^{K-1}\beta_{k}\Lambda^{k}$$

where *K* denotes the maximum order of the polynomial, with *K* being substantially smaller than 21. This polynomial approximation reduces the number of convolution kernel parameters from *n* to *K*. Nevertheless, since the input signal still requires multiplication by the matrix *U*, the computational complexity of the graph convolution operation remains at O(*n*^2^). Therefore, Chebyshev polynomials are adopted, and the convolution operation is termed Chebyshev convolution [39]. The convolution kernel can be transformed as follows:

(16) $$g_{\theta }\left(\Lambda \right)=\sum_{k=0}^{K-1}\beta_{k}T_{k}(\hat{\Lambda })$$

where $\beta_{k}$ denotes the Chebyshev polynomial coefficient, and $T_{k}(\hat{\Lambda })$ represents the Chebyshev polynomial evaluated at the scaled Laplacian matrix $\hat{\Lambda }=2\Lambda /\lambda_{\max}-I_{n}$. In the implementation of graph convolution computations, the Chebyshev polynomial can be formulated as follows:

(17) $$\begin{array}{l} T_{0}(L)=I\\ T_{1}(L)=L\\ T_{k}(L)=2LT_{k-1}(L)-T_{k-2}(L) \end{array}$$

Subsequent to polynomial fitting, the convolution operation can be rephrased as follows:

(18) $$x{*}_{G}g_{\theta }=Ug_{\theta }(\Lambda )U^{T}x=U(\sum_{k=0}^{K-1}\beta_{k}T_{k}(\hat{\Lambda }))U^{T}x=\sum_{k=0}^{K-1}\beta_{k}T_{k}(U\hat{\Lambda }U^{T})x=\sum_{k=0}^{K-1}\beta_{k}T_{k}(\hat{L})x$$

where $\hat{L}=2L/\lambda_{\max}-I_{n}$, and $\lambda_{\max}=2$ are defined as above. With the adoption of this strategy, eigendecomposition of the Laplacian matrix is no longer necessary, and the computational complexity is reduced from O(*n*^2^) to O(*kn*). Concurrently, benefiting from the polynomial-based calculation paradigm, the number of convolution parameters is diminished from *n* to *k*. In the present study, the parameter *k* was set to 2.

(4) WGCNet Architecture.

Based on the computational procedures described above, WGCNet was designed (Pseudocode shown in Algorithm 1). First, two graph convolutional layers (GC) were employed to extract deep EEG features. To balance computational complexity and feature-extraction effectiveness, preliminary experiments determine that each graph convolution used 20 kernels. Subsequently, a fast graph pooling operation was applied to aggregate and reduce the dimensionality of the deep EEG features. Finally, two fully connected layers (FC) performed nonlinear mapping from the deep EEG features to the labels corresponding to different taste concentrations. The first FC layer contained 32 neurons, and the second FC layer contained 6 neurons.
**Algorithm 1:** Wavelet Graph Convolutional Neural Network (WGCNet)Input: EEG signals: $X\in {ℜ}^{N\times C\times T}$. (*N* = samples, *C* = 21 channels, *T* = 256 timepoints); Num_classes = 6Output: Classification probability: $P\in {ℜ}^{N\times 6}$
1. For each sample x∈X: a. Morlet wavelet: Decompose each channel into Delta/Theta/Alpha/Beta/Gamma bands b. Compute for each band: Instant energy E, then $E_{\max}=\max(E)$, $E_{\mathrm{mean}}=\mathrm{mean}(E)$
 c. Channel feature: $f_{c}=\left[E_{\max,1},E_{\mathrm{mean},1},\cdots ,E_{\max,5},E_{\mathrm{mean},5}\right]\in {ℜ}^{10}$
 d. Sample feature matrix: $F=\left[f_{1},f_{2},\cdots ,f_{21}\right]\in {ℜ}^{21\times 10}$
2. Build graph: Nodes = *F*; Adjacency matrix A=PearsonCorr(F)
3. Chebyshev GCN (*K* = 2): a. Laplacian L=D−A
 b. Chebyshev polynomials: $T_{0}=I,T_{1}=L$
 c. GConv1: 20 kernels d. GConv2: 20 kernels4. Pooling + FC: a. Pooling = FastAveragePooling (GConv2) b. FC1: 32 neurons c. FC2: 6 neurons5. Return P


### 2.4. Cross-Subject Training Based on Meta-Learning

The core goal of the meta-learning is to enable the model to “learn how to learn”, rather than directly learning the EEG features of a specific group of subjects. This aims to solve the problem of sharp accuracy degradation in cross-subject recognition caused by individual brain differences. Traditional deep learning is “trained on a batch of subjects and applied directly to new subjects”, which often fails due to distribution differences across individuals. In contrast, meta-learning extracts general taste-related neural representations from multiple subjects and learns a set of “good initialization parameters”. This allows the model to quickly adapt to a completely new subject with only a small number of samples and a few gradient update steps. The technical process is as follows:

Step 1: Data Construction.

A total of 10,800 samples (20 subjects × 6 classes × 90 samples per class) are partitioned by subject into 20 independent “task domains”. The data from each subject constitutes a distinct meta-task, within which support and query sets are defined. For each subject, 30 samples per class are randomly selected to form the support set (following a 6-way 30-shot task structure), while the remaining 60 samples per class serve as the query set. The support set is used for meta-model training to enable rapid learning, and the query set is used for evaluation and meta-loss computation. Let the overall dataset be denoted as $D=\{D_{1},D_{2},\cdots ,D_{20}\}$, where $D_{i}$ represents the EEG data of the *i*-th subject. Each $D_{i}$ comprises six concentration-level labels (including a control condition). For each subject dataset $D_{i}$, the support set $S_{i}$ and query set $Q_{i}$ are partitioned according to the “6-way 30-shot” scheme. The meta-task $T_{i}=(S_{i},Q_{i})$ is thus constructed accordingly.

Step 2: Meta-learning Training and Cross-Validation.

(1) Outer-Loop Leave-One-Subject-Out Validation: A rigorous cross-subject evaluation is performed using the leave-one-subject-out method. Specifically, the data from one subject are held out as the meta-test task (representing a completely unseen subject), while the data from the remaining 19 subjects serve as meta-training tasks.

(2) Inner-Loop Meta-Cross-Training: Among the 19 meta-training task domains, a five-fold cross-validation scheme (4, 4, 4, 4, 3) is adopted to partition the meta-training and meta-validation sets. The meta-learning model, which employs model-agnostic meta-learning (MAML), learns a good initial parameter set directly, enabling rapid adaptation to a new subject with only a few gradient steps. This model iteratively trains the WGCNet on the meta-training set. The core process is as follows: For each meta-task, a few gradient update steps are performed on its support set (rapid adaptation), after which the loss is computed on the query set. The gradients are then aggregated to update the initialization parameters of the meta-model. The objective is to enable the model to learn how to quickly adapt to a new subject. The meta-validation set is used for model parameter tuning and identifying the optimal classification network. Let the initial parameters of the meta-model be denoted as θ. For each meta-training task *T*, rapid task-specific adaptation is first carried out using its support set *S*. The updated task parameters, denoted as $\theta^{\prime }$, are defined as follows:

(19) $$\theta^{\prime }=\theta -\eta_{task}\nabla_{\theta }L(M(\theta ;S),y_{S})$$

where $\eta_{task}$ is the within-task learning rate (set to 0.01); *L* denotes the *L*2 cross-entropy loss function (the coefficient of the penalty term is 0.01). M(θ;S) represents the predicted output of the WGCNet model with parameters θ for the support set *S*, and $y_{S}$ denotes the ground-truth labels of the support set *S*. A multi-step adaptation strategy (default: 3 steps) is employed during training, meaning that $\theta^{\prime }$ undergoes multiple iterative updates:

(20) $$\theta^{k}=\theta^{k-1}-\eta_{task}\nabla_{\theta^{k-1}}L(M(\theta^{k-1};S),y_{S}),\begin{array}{cc} & \theta^{\prime }=\theta^{3} \end{array}$$

The meta-loss function is defined as the average loss over the query sets of all meta-training tasks under the adapted parameters $\theta^{\prime }$:

(21) $$L_{meta}(\theta )=\frac{1}{\left|T_{Train}\right|}\sum_{T\in T_{Train}}L(M(\theta^{\prime };Q),y_{Q})$$

where $T_{train}$ denotes a subset of meta-training tasks, $y_{Q}$ represents the ground-truth labels of the query set *Q*, and $M(\theta^{\prime };Q)$ is the predicted output of the model on the query set after its parameters are updated to $\theta^{\prime }$. The meta-parameters θ are updated via gradient descent on the meta-loss, following the update rule:

(22) $$\theta \leftarrow \theta -\eta_{meta}\nabla_{\theta }L_{meta}(\theta )$$

where $\eta_{meta}$ represents the meta-learning rate, set to 0.01.

Step 3: Testing and Evaluation.

When the trained meta-model encounters a test subject, the data of that subject are defined as a meta-test task $T_{test}=(S_{test},Q_{test})$. Using the 30 samples per class from this subject as the support set $S_{test}$, rapid adaptation is performed. The adapted parameters for the new task are obtained as follows:

(23) $$\theta_{adapt}=\theta_{best}-\eta_{task}\nabla_{\theta_{best}}L(M(\theta_{best};S_{test}),y_{test})$$

The classification performance is then evaluated using the query set $Q_{test}$ of that subject. This process is repeated 20 times, each time leaving out a different subject as the test domain. Finally, the average performance over all 20 runs is computed as the final metric for cross-subject generalization capability. The performance evaluation indicators of the leave-one-out method include accuracy, precision, recall rate, and F1-score [40,41].

## 3. Results and Discussion

### 3.1. Spatial Response Patterns of Brain Regions Under Gustatory EEG Stimulation

sLORETA (Standardized Low-Resolution Brain Electromagnetic Tomography) is a source localization analysis method for EEG data; it obtains a standardized solution for current density distribution and theoretically achieves unbiased source localization under the constraint of zero localization error [42]. Based on the sLORETA method, source localization analysis was performed on EEG signals evoked by taste stimuli of different sweet and bitter concentrations, in order to physiologically prove that different concentrations of sweet and bitter tastes indeed induce distinguishable cortical activation patterns, and to provide a physiological interpretability basis for the model’s design. This ensured that the entire framework not only achieved “good performance” but was also “consistent with brain mechanisms”.

(1) Spatial Distribution Changes in Sweet-Taste Activation under Different Sweet-Taste Concentration Conditions.

**Figure 5:**
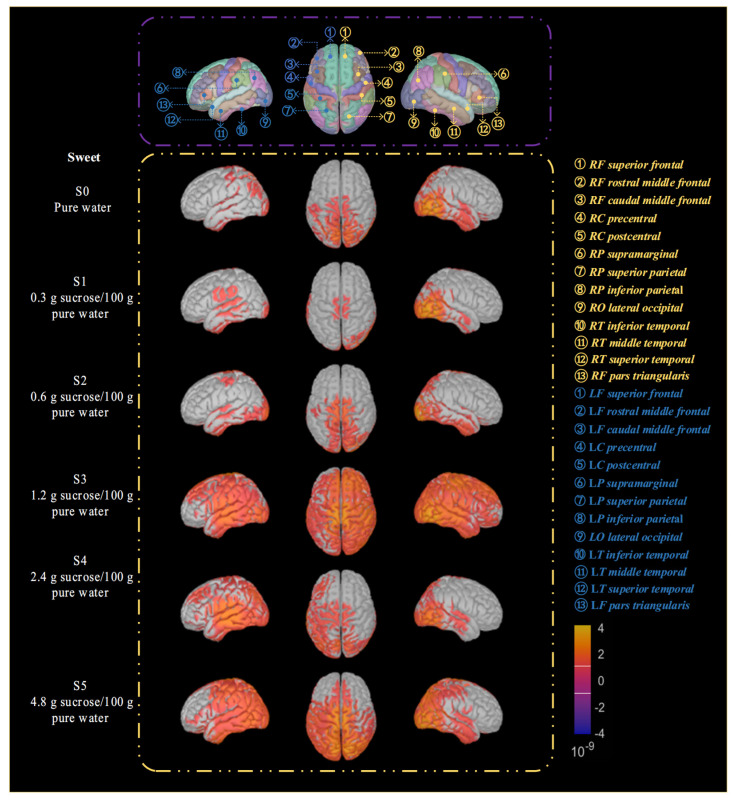
Changes in brain region activation under different concentrations of sweet stimulation.

Figure 5 shows the changes in brain region activation under different sweet concentrations. From a spatial distribution perspective, the brain activation patterns under different sweet-taste concentrations exhibit clear stage-wise differences. Under the S 0 condition, cortical activation is generally weak and relatively limited in spatial distribution, mainly localized in cortical areas such as the postcentral gyrus and supramarginal gyrus, which are associated with oral somatosensory input and multimodal sensory integration. This activation pattern primarily reflects nonspecific oral sensation and basic somatosensory processing.

As the sweet-taste concentration increases (S 1 to S 2), activated regions gradually concentrate in cortices that are typically involved in taste processing. From three viewing angles (left, top, and right), activation is mainly observed in the bilateral insula and its adjacent cortical areas, accompanied by stable involvement of regions such as the inferior frontal gyrus and inferior parietal lobule. Overall, activation at this stage remains dominated by sensory and parietal-related areas, with a relatively limited spatial extent that has not yet clearly extended to higher-order cognitive regions in the prefrontal cortex. This suggests that low-concentration sweet-taste stimuli primarily elicit primary sensory coding of taste and its synergistic processing with parietal sensation. Under medium sweet-taste concentration conditions (S 3), the spatial distribution of cortical activation shows evident changes. In addition to sustained significant activation in the insula and parietal-related areas, activated regions begin to extend into the prefrontal cortex, with involvement observed in areas such as the middle frontal gyrus and superior frontal gyrus. At this point, the activation pattern evolves from a relatively focal sensory-related distribution to a distributed network encompassing sensory, parietal, and partial prefrontal regions, indicating that taste processing has transitioned from a purely sensory coding stage to a stage involving attentional allocation and primary cognitive evaluation.

Under high sweet-taste concentration conditions (S 4 to S 5), the extent of cortical activation reaches its maximum, with activated regions significantly expanding and exhibiting relatively symmetric bilateral distribution characteristics. In addition to high-intensity activation maintained in the bilateral insula, postcentral gyrus, and parietal-related areas, multiple prefrontal regions (including the superior frontal gyrus, middle frontal gyrus, and triangular part of the inferior frontal gyrus) show clear involvement. Concurrently, the synergistic activation of the superior parietal lobule and inferior parietal lobule becomes more prominent. This spatial distribution pattern indicates that under high-intensity sweet-taste stimulation, taste processing is no longer confined to sensory-related cortices but forms a distributed cortical network encompassing sensory processing, attention, cognitive evaluation, and potential reward-value processing.

(2) Changes in the Spatial Distribution of Brain Activation under Different Bitter-Taste Concentration Conditions.

**Figure 6:**
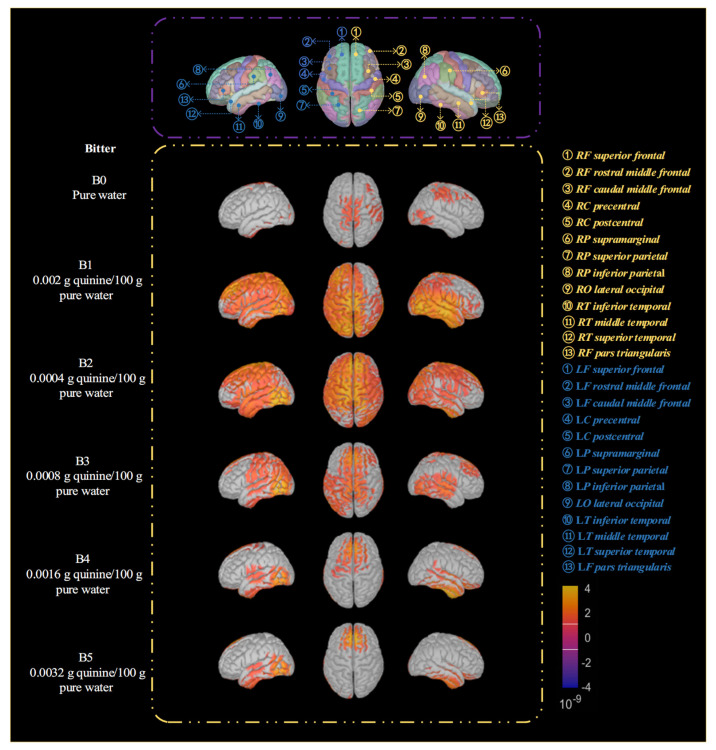
Changes in brain region activation under different concentrations of bitter stimulation.

Figure 6 illustrates the changes in brain region activation under different bitter-taste concentration stimulations. The spatial distribution of cerebral cortex activation exhibits clear and stage-wise variation characteristics across different bitter-taste concentrations. Under low-concentration conditions, cortical activation is primarily concentrated in the insula and prefrontal cortex. Among these, the insula, as a core node of the primary gustatory cortex, undertakes the functions of basic perception and intensity coding of bitter-taste stimuli. The early involvement of the prefrontal cortex suggests that even at low concentrations, bitter-taste stimuli already initiate a certain degree of alertness processing. As the stimulus concentration increases to a medium level, the activation range expands significantly, gradually covering the parietal association area and the anterior cingulate cortex. The participation of the parietal association area reflects the enhancement of multimodal sensory integration and attentional resource allocation. The activation of the anterior cingulate cortex, an important node for emotion and cognitive control, indicates that bitter-taste processing at this stage is no longer confined to the sensory level. Under high-concentration conditions, cortical activation further extends to the temporal lobe and anterior frontal regions, including the orbitofrontal cortex and dorsolateral prefrontal cortex. Among these, the orbitofrontal cortex is typically associated with taste value evaluation and reward/punishment judgment, while the involvement of the dorsolateral prefrontal.

Cortex reflects higher-order cognitive control and decision-making processes. This spatial distribution pattern demonstrates that high-intensity bitter-taste stimuli are not only perceived as unpleasant experiences but also significantly engage cortical networks related to risk assessment and behavioral regulation. However, under extremely high-concentration conditions, the spatial distribution of cortical activation shows a contracting trend, with activation mainly confined to core regions such as the insula and anterior cingulate cortex, which are closely associated with aversive experience, alertness response, and defensive regulation. Compared to the high-concentration stage, this suggests that under overly intense stimulation, the brain may respond to strong negative input by selectively recruiting key nodes rather than continuously expanding the activation range.

It is evident that the spatial activation patterns of the brain change markedly under stimulation with different sweet-taste and bitter-taste concentrations, demonstrating the feasibility of achieving precise identification of sweet-taste and bitter-taste concentrations using EEG signals. Meanwhile, due to individual differences, cross-subject recognition research becomes a critical issue in EEG-based identification under the premise of achieving precise discrimination. Furthermore, under each experimental condition, we averaged the current density values back-sourced from all brain regions. Then, paired *t*-tests were conducted to compare the resulting t-values, *p*-values, and significance levels between different groups, in order to demonstrate the statistical significance of the brain activation patterns. The analytical results were presented in Table S2 of the Supplementary File.

### 3.2. Ablation Experiments of WGCNet

Table 1 and Table 2 present the results of the ablation experiments conducted on the cross-subject ML-WGCNet under stimulation with different sweet and bitter concentrations, respectively. The core objective was to validate the independent contributions and synergistic effects of each key model component on classification performance. Using a controlled variable method, the experiments compared the accuracy, precision, recall, and F1-score of the model under different configurations by progressively adding or removing individual components. This approach clarifies the necessity of each component.

The experimental results for both taste stimuli show that when only the GCN is included, the model’s classification performance is at its lowest level, although the overall cross-subject classification performance remains acceptable. This result indicates that while the GCN, as the foundational framework of the classification network, can extract partial discriminative information by establishing spatial correlations among electrodes, relying solely on the spatial structural features of the raw EEG signal without capturing key information in the time–frequency domain leads to limited classification performance. Additionally, the standard deviation is larger for bitter-taste stimulation, suggesting that in the absence of other feature-enhancement components, the model exhibits poorer classification stability for bitter-taste EEG signals, which may be related to the higher variability of neural responses elicited by bitter-taste stimuli. Upon adding wavelet decomposition to the GCN, the classification performance for both taste types improves significantly. This improvement confirms the core value of wavelet decomposition: EEG signals possess multi-scale rhythmic characteristics, and the time–frequency decomposition using the Morlet wavelet transforms the time-domain signal into a two-dimensional feature matrix containing five frequency bands (Delta, Theta, Alpha, Beta, Gamma), effectively isolating the neural rhythmic responses evoked by taste stimulation. Comparing the degree of improvement between the two taste types, the accuracy improvement rate for bitter taste is higher than that for sweet taste, indicating that wavelet decomposition provides a more substantial gain for bitter-taste EEG signals. This may be because the neural responses to bitter-taste stimuli exhibit more pronounced differences in the frequency domain, and wavelet decomposition precisely captures these differences.

Adding the mean wavelet energy on top of the “wavelet decomposition + GCN” combination further enhances the classification performance for both sweet and bitter tastes. The mean wavelet energy reflects the average level of neural activity in each frequency band over the entire stimulation period; it characterizes the overall activation trend of neural rhythms during taste perception and supplements the global feature information that is lacking after time–frequency decomposition, thereby strengthening the model’s ability to distinguish between different concentration stimuli. When the maximum wavelet energy is added to the “wavelet decomposition + GCN” combination, the model performance improvement is even more pronounced. The maximum wavelet energy can highlight the peak neural responses evoked by taste stimuli, capturing the most prominent rhythmic activity features and reflecting the intensity of key activation moments during neural processing. This instantaneous feature exhibits higher sensitivity to different concentration stimuli, which is why its contribution to classification performance surpasses that of the energy mean. When both the mean and maximum wavelet energy are added simultaneously, the model achieves its optimal performance. This result demonstrates that the mean energy (global trend) and the maximum energy (instantaneous peak) are complementary feature dimensions; their combination comprehensively characterizes the non-stationary properties of EEG signals, reflecting both the overall neural activity level throughout the stimulation process and the intensity differences at critical activation moments, thereby providing richer discriminative information for the classification model.

The ablation experiments in Table 1 and Table 2 clearly reveal the functional value and synergistic mechanisms of the components in WGCNet. Wavelet decomposition lays the foundation for feature extraction. The mean and maximum wavelet energy provide complementary information on global trends and instantaneous peaks, respectively. The GCN models the spatial correlations among electrodes. The synergy of these four components enables efficient classification of taste-related EEG signals. This result validates the rationality of the model architecture.

### 3.3. Cross-Subject EEG Classification Results

Table 3 presents the cross-subject EEG classification results of the ML-GCNet model under different sweet concentrations, while Table 4 shows its corresponding results under different bitter concentrations. Based on the experimental design and the neural response patterns of taste, a detailed analysis and discussion are provided regarding the overall classification performance, individual differences, and concentration gradient effects.

**Figure 7:**
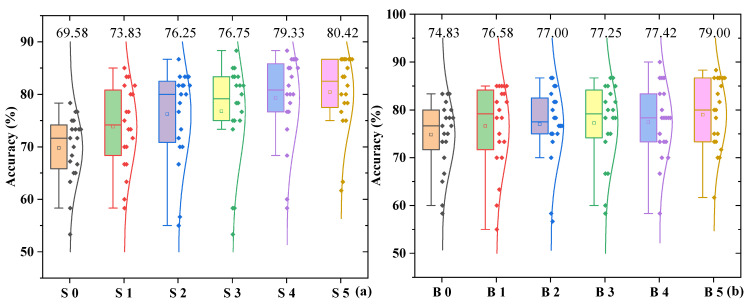
The overall classification performance comparison results of cross-subjects. (**a**) Sweetness stimulation, (**b**) Bitterness stimulation.

From the overall performance of classification across sweet-taste concentrations shown in Figure 7a, a clear gradient-increasing trend is observed. This result indicates that the model’s classification ability for EEG signals progressively strengthens with increasing sweet concentration. The primary reason for this is that low-concentration sweet-taste stimuli elicit weaker neural responses, resulting in less distinct physiological differences compared to the pure-water control and fewer specific features in the EEG signals. In contrast, high-concentration sweet-taste stimuli activate a broader cortical network (as described in Section 3.1, where the bilateral insula, prefrontal, parietal, and other regions were co-activated under high concentration), leading to more pronounced intensity and time–frequency characteristics in neural responses, which provide richer discriminative information for the model. The overall increase in classification performance across bitter concentrations shown in Figure 7b is relatively moderate, with little variation, which is related to the neurophysiological mechanisms of bitter taste. Even low-concentration bitter-taste stimuli can rapidly activate the insula (primary gustatory cortex) and alertness-related processing regions in the prefrontal cortex (as shown in Section 3.1), making their difference from the pure-water control more significant. Therefore, the classification accuracy for B 0 is much higher than that for S 0. Under high-concentration bitter-taste stimuli, although the cortical activation range expands, it does not exhibit the significant leap observed in sweet-taste responses, resulting in relatively stable performance differences across concentration gradients. Based on the average classification accuracy for each concentration in Table 3 and Table 4, the cross-subject classification average accuracy for bitter taste is 77.01%, slightly higher than the 76.03% for sweet-taste classification. This indicates that the model has a slight advantage in cross-subject recognition of bitter-taste EEG signals.

Table 3 reveals significant individual differences in sweet-taste classification accuracy among different subjects. The highest classification performance was observed in Subject 16 (83.89%), while the lowest was in Subject 15 (57.22%), resulting in a difference of 26.67%. Specifically, the high-performance group (accuracy ≥ 80%) includes Subjects 2, 3, 5, 7, 9, 11, 12, 16, and 18, totaling nine subjects, which accounts for 45% of the participants. For these subjects, the time–frequency characteristics of their EEG signals exhibit more distinct variations across different sweet-taste concentrations, which may be related to their high individual taste sensitivity and the regularity of neural activity in the cerebral cortex. The low-performance group (accuracy ≤ 60%) consists only of Subjects 4 and 15, accounting for 10% of the participants. Possible reasons for this include higher individual taste thresholds where low-concentration stimuli fail to elicit significant neural responses or factors related to the subjects’ attention levels and physiological fluctuations during the experiment. The moderate-performance group (60% < accuracy < 80%) comprises the remaining nine subjects (45%), whose classification performance is relatively stable, with smaller fluctuations in accuracy across concentrations, indicating better adaptability of the model for this group. Table 4 shows that individual differences also exist in bitter-taste classification, but the overall fluctuation range is smaller than that for sweet-taste classification. The highest accuracy was observed in Subjects 7 and 9, while the lowest was in Subject 5, with a difference of 26.39%. The high-performance group (accuracy ≥ 80%) includes Subjects 4, 6, 7, 9, 10, 14, 15, 17, 18, and 20, totaling 10 subjects, accounting for 50% of the participants, which is higher than the proportion in the sweet-taste high-performance group. The low-performance group (accuracy ≤ 60%) consists only of Subject 5, accounting for 5% of the participants, which is lower than the proportion in the sweet-taste low-performance group. The moderate-performance group (60% < accuracy < 80%) includes nine subjects (45%). The individual stability in bitter-taste classification is better, possibly because bitter taste, as an “alerting taste”, relies on neural processing pathways that are more innate and consistent, and is less influenced by individual dietary preferences or physiological states compared to sweet taste.

In Table 3, the relationship between sweet-taste concentration and classification accuracy is not simply linear but exhibits a nonlinear pattern characterized by “rapid improvement at low concentrations, stabilization at medium concentrations, and further improvement at high concentrations”. The transition from “no sweet taste (pure water)” to “low sweet taste” shows a significant difference, as the time–frequency characteristics of EEG signals undergo qualitative changes, making it easier for the model to distinguish them. Under low-to-medium sweet-taste concentrations, cortical activation primarily concentrates in primary sensory areas such as the insula and parietal lobes (as shown in Section 3.1), leading to smaller differences in neural response patterns and thus increasing the difficulty of classification for the model. At high concentrations, higher-order cognitive regions such as the prefrontal cortex become involved in taste processing, forming a distributed cortical network. This further expands the differences in the spatial correlation and time–frequency characteristics of EEG signals, providing more basis for classification. In Table 4, the relationship between bitter-taste concentration and classification accuracy is relatively moderate. The increase from “no bitter taste (pure water)” to “low bitter taste” is much smaller than the increase observed in the same concentration range for sweet taste, because low-concentration bitter-taste stimuli can already activate alertness-related processing pathways, resulting in relatively clear differences from pure water. Therefore, further increases in concentration provide limited additional feature gains. The increase in accuracy gradually becomes more pronounced across concentrations, a feature related to the neural processing mechanisms of bitter taste. Bitter-taste stimuli at medium concentrations or above can sufficiently activate the relevant cortical network. Although further increases in concentration enhance the intensity of neural responses, they do not lead to significant expansion of the processing pathways (as mentioned in Section 3.1, the activation range contracts after high concentrations), resulting in a moderate improvement in the model’s classification performance.

To demonstrate the superiority of ML-GCNet in cross-subject classification, we compared it with state-of-the-art cross-subject EEG classification methods, which are described in the Introduction. All comparative experiments followed the principles of using the same dataset, the same preprocessing, the same evaluation metrics, and the same data partitioning strategy. Table 5 presents the performance comparison between the ML-WGCNet model and the state-of-the-art cross-subject EEG classification methods under different sweetness concentrations. The results clearly show that our model significantly outperforms existing methods across all evaluation metrics. Table 6 illustrates the cross-subject classification performance of ML-WGCNet under different bitterness concentrations; its overall performance is consistent with that in sweetness classification, with even greater advantages on certain metrics. This benefits from the synergistic strategy of the model, which integrates wavelet time–frequency feature extraction and graph convolutional spatial modeling, enabling a more comprehensive capture of the dynamic changes in EEG signals and inter-regional brain connectivity under sweet taste stimulation. Furthermore, the relatively small standard deviation of the model’s results indicates its more stable generalization ability in cross-subject scenarios, effectively alleviating performance fluctuations caused by individual differences. A comparison between Table 5 and Table 6 reveals that the average performance of ML-WGCNet in bitterness classification is slightly higher than that in sweetness classification. This may stem from the fact that the neural processing pathways of bitterness, as a “warning taste”, are more innate and consistent, and are less influenced by factors such as individual dietary preferences. The meta-learning training framework of the model further amplifies this advantage, enabling efficient adaptation to cross-subject recognition of different taste types.

Table 7 shows a comparison of computational complexity between ML-WGCNet and state-of-the-art EEG cross-subject methods, evaluated in terms of the number of parameters (Params) and floating-point operations (FLOPs). While achieving optimal performance, ML-WGCNet also exhibits lightweight structural properties, further facilitating its practical deployment in human–computer interaction engineering. To further demonstrate the statistical validity of the results obtained by ML-WGCNet, we conducted a statistical test analysis. All compared algorithms in this study were tested under the same data partitioning, the same taste EEG dataset, and the same leave-one-subject-out cross-validation scheme. This constitutes a typical paired design, meeting the applicable conditions for the paired *t*-test. At the same time, the paired *t*-test effectively controls for confounding factors such as individual differences among subjects and experimental conditions, allowing for a more accurate evaluation of whether the performance differences between different models are statistically significant. Table 8 presents the statistical test results on two different datasets. We repeated the training and testing process 50 times, performed statistical analysis on the 50 sets of results, and obtained the t-values, *p*-values, and significance levels. The analysis results show that ML-WGCNet achieves significant differences compared with other cross-subject EEG data classification methods.

### 3.4. Research Limitations and Future Work

This paper uses sLORETA to qualitatively analyze brain activation patterns under different taste concentrations, thereby providing a physiological basis for cross-subject EEG classification. Then, ML-WGCNet is employed to achieve cross-subject EEG taste recognition. This research paradigm holds significant application potential and can be extended to areas such as motor imagery [33], epilepsy monitoring [43], and emotion recognition [44]. Although our proposed method achieves better performance in cross-subject sweet- and bitter-taste EEG classification, there are needs that need to be gradually addressed in future work:

(1) High Homogeneity of Participant Samples: To provide a research paradigm and a technical method for subsequent cross-individual EEG taste studies, this study only recruited 20 healthy young participants, aged 20–24 years. However, the narrow age range and limited demographic diversity mean that the model’s generalizability across different ages, genders, and populations has not been fully validated.

(2) Limited Types of Taste Stimuli: To validate the effectiveness and discriminative ability of a novel EEG recognition model, this study selected sweet and bitter tastes, which show clear differences in cortical activation patterns. Nevertheless, other common tastes, such as sour, salty, and umami, were not included, nor were mixed flavors.

(3) Insufficient Representational Richness of EEG Channels: This experiment used a 21-channel EEG device, which can cover the main brain regions involved in taste perception, and established an EEG-based cross-subject classification method. However, the spatial resolution was limited. More detailed classification of brain activation patterns in source space, as well as the performance transferability to high-density montages, requires further validation.

Based on the above limitations, future research can be carried out in the following three directions.

(1) Expand Sample Diversity: To improve the cross-population generalizability of the model, future studies can continuously recruit participants of different ages, genders, and regional backgrounds in order to build a more representative dataset. This will comprehensively enhance the model’s generalizability and robustness across different populations.

(2) Expand Taste Types: To improve the multi-flavor recognition system, basic taste stimuli such as sour, salty, and umami can be added. Datasets containing mixed tastes and real food flavors can also be constructed, in order to form an EEG taste recognition method covering multiple types and complex scenarios.

(3) Combine High-Density EEG with Source-Space Features: To optimize modeling accuracy and interpretability, high-density EEG devices can be used to improve source localization precision. Source-space features can also be integrated into the classification model in order to further enhance classification performance and physiological plausibility and to promote the translation of this method toward practical engineering applications.

## 4. Conclusions

This study addresses the problem of cross-subject EEG recognition for sweet and bitter tastes by constructing an integrated technical framework combining wavelet time–frequency analysis, graph convolutional modeling, and meta-learning training. The core conclusions are as follows:

(1) EEG signals under taste stimulation exhibit significant time–frequency rhythmic characteristics and spatial distribution patterns. With increasing concentration of sweet taste stimulation, brain activation progressively extends from primary sensory regions such as the postcentral gyrus and insula to a distributed cognitive network encompassing the prefrontal and parietal lobes. Bitter taste stimulation shows insula-prefrontal alert-related activation at low concentrations and focused activation in core regions (insula, anterior cingulate cortex) at high concentrations. The distinct neural response patterns between the two taste types provide a reliable physiological basis for discriminating concentration.

(2) The collaborative feature extraction strategy combining wavelet transform and graph convolution effectively enhances the discriminability of taste-related EEG. Morlet wavelet decomposition accurately captures neural rhythmic responses across different frequency bands. The combined features of maximum energy (instantaneous peaks) and average energy (global trends) comprehensively characterize the non-stationary properties of EEG signals. GCN further mines cooperative processing information among brain regions by modeling spatial relationships between electrodes. Ablation experiments confirmed that the combination of the four elements (wavelet decomposition + average energy + maximum energy + GCN) constitutes the optimal model configuration.

(3) The meta-learning framework significantly improves the generalization performance for cross-subject classification. By constructing data from individual subjects as independent meta-tasks and employing leave-one-subject-out cross-validation with rapid adaptation training, average accuracies of 76.03% and 77.01% were achieved in cross-subject tests across 20 subjects for sweet- and bitter-taste classification, respectively. The corresponding precision values are 79.94% and 79.53%, the recall values are 75.77% and 78.51%, and the F1-scores are 78.24% and 78.08%, respectively.

This study establishes an effective method for cross-subject EEG taste recognition, overcoming the limitations of traditional evaluation approaches and providing technical support for the objective, standardized, and personalized assessment of flavor.

## Figures and Tables

**Table 1:** Analysis results of ML-WGCNet ablation experiments under different sweet concentrations.

WD	WEMean	WEMax	GCN	Accuracy (%)	Precision (%)	Recall (%)	F1-Score (%)
-	-	-	√	63.02 ± 8.86	64.39 ± 9.32	62.84 ± 8.71	63.73 ± 8.69
√	-	-	√	66.60 ± 7.13	68.46 ± 8.74	66.85 ± 7.06	67.59 ± 7.74
√	√	-	√	70.52 ± 6.91	72.77 ± 7.76	70.75 ± 7.83	71.70 ± 7.58
√	-	√	√	73.35 ± 7.20	74.59 ± 7.54	73.43 ± 7.10	73.57 ± 6.43
√	√	√	√	76.03 ± 7.46	79.94 ± 7.44	75.77 ± 7.27	78.24 ± 7.34

Note: WD = wavelet decomposition, WEMean = mean wavelet energy, WEMax = maximum wavelet energy.

**Table 2:** Analysis results of ML-WGCNet ablation experiments under different bitter concentrations.

WD	WEMean	WEMax	GCN	Accuracy (%)	Precision (%)	Recall (%)	F1-Score (%)
-	-	-	√	61.19 ± 11.24	61.44 ± 10.84	61.05 ± 11.00	61.34 ± 9.88
√	-	-	√	67.65 ± 7.22	69.07 ± 7.91	67.37 ± 8.20	68.35 ± 8.75
√	√	-	√	71.93 ± 8.28	73.16 ± 9.99	73.03 ± 7.89	73.00 ± 8.63
√	-	√	√	72.27 ± 6.22	73.43 ± 7.88	72.80 ± 6.04	72.96 ± 6.62
√	√	√	√	77.01 ± 7.22	79.53 ± 8.13	78.51 ± 7.37	78.08 ± 7.44

**Table 3:** Classification results of cross-subject EEG under different sweet concentrations with ML-WGCNet.

Subject	S 0 (%)	S 1 (%)	S 2 (%)	S 3 (%)	S 4 (%)	S 5 (%)	Mean Accuracy (%)
No. 1	63.33	70.00	76.67	75.00	80.00	80.00	74.17
No. 2	71.67	75.00	81.67	83.33	88.33	86.67	81.11
No. 3	73.33	81.67	83.33	83.33	85.00	86.67	82.22
No. 4	58.33	60.00	55.00	58.33	60.00	61.67	58.89
No. 5	78.33	83.33	80.00	81.67	85.00	83.33	81.94
No. 6	68.33	70.00	66.67	73.33	76.67	76.67	71.94
No. 7	75.00	83.33	86.67	85.00	81.67	86.67	83.06
No. 8	70.00	66.67	71.67	75.00	73.33	75.00	71.94
No. 9	75.00	73.33	81.67	83.33	86.67	86.67	81.11
No. 10	73.33	76.67	81.67	78.33	80.00	83.33	78.89
No. 11	65.00	80.00	83.33	85.00	86.67	85.00	80.83
No. 12	71.67	80.00	80.00	81.67	86.67	86.67	81.11
No. 13	65.00	66.67	70.00	78.33	83.33	78.33	73.61
No. 14	76.67	76.67	73.33	80.00	76.67	80.00	77.22
No. 15	53.33	58.33	56.67	53.33	58.33	63.33	57.22
No. 16	73.33	85.00	83.33	88.33	86.67	86.67	83.89
No. 17	66.67	63.33	70.00	58.33	68.33	75.00	66.94
No. 18	76.67	81.67	83.33	81.67	85.00	86.67	82.50
No. 19	63.33	73.33	78.33	75.00	80.00	78.33	74.72
No. 20	73.33	71.67	81.67	76.67	78.33	81.67	77.22
Mean Accuracy (%)	69.58	73.83	76.25	76.75	79.33	80.42	76.03

**Table 4:** Classification results of cross-subject EEG under different bitter concentrations with ML-WGCNet.

Subject	B 0 (%)	B 1 (%)	B 2 (%)	B 3 (%)	B 4 (%)	B 5 (%)	Mean Accuracy (%)
No. 1	65.00	60.00	70.00	66.67	73.33	73.33	68.06
No. 2	66.67	70.00	75.00	66.67	70.00	75.00	70.56
No. 3	76.67	81.67	75.00	81.67	75.00	73.33	77.22
No. 4	83.33	80.00	83.33	78.33	78.33	80.00	80.56
No. 5	58.33	55.00	58.33	60.00	58.33	61.67	58.61
No. 6	75.00	83.33	78.33	80.00	86.67	78.33	80.28
No. 7	80.00	85.00	86.67	85.00	86.67	86.67	85.00
No. 8	73.33	73.33	78.33	75.00	73.33	80.00	75.56
No. 9	81.67	85.00	86.67	85.00	83.33	88.33	85.00
No. 10	78.33	85.00	85.00	83.33	86.67	86.67	84.17
No. 11	60.00	63.33	56.67	58.33	66.67	70.00	62.50
No. 12	75.00	78.33	73.33	75.00	78.33	83.33	77.22
No. 13	70.00	70.00	75.00	73.33	78.33	70.00	72.78
No. 14	83.33	85.00	80.00	86.67	80.00	86.67	83.61
No. 15	78.33	75.00	81.67	80.00	83.33	85.00	80.56
No. 16	76.67	83.33	85.00	78.33	73.33	75.00	78.61
No. 17	83.33	78.33	81.67	86.67	78.33	86.67	82.50
No. 18	80.00	85.00	76.67	81.67	90.00	86.67	83.33
No. 19	73.33	73.33	76.67	78.33	70.00	71.67	73.89
No. 20	78.33	81.67	76.67	85.00	78.33	81.67	80.28
Mean Accuracy (%)	74.83	76.58	77.00	77.25	77.42	79.00	77.01

**Table 5:** Comparison results of ML-WGCNet with the state-of-the-art cross-subject EEG classification methods under different sweet concentrations.

Methods	Accuracy (%)	Precision (%)	Recall (%)	F1-Score (%)
MTL [30]	65.23 ± 8.02	64.13 ± 8.44	67.42 ± 9.80	65.46 ± 9.24
DDA [21]	66.79 ± 8.67	68.19 ± 9.67	67.75 ± 8.08	67.82 ± 8.98
EPEL [23]	67.77 ± 11.63	69.10 ± 12.75	68.41 ± 11.30	68.52 ± 11.63
WBT [22]	70.23 ± 7.23	72.50 ± 7.81	70.88 ± 8.21	71.33 ± 8.04
MC [29]	70.62 ± 7.23	72.50 ± 7.87	70.88 ± 8.21	71.33 ± 8.04
CDSCN [27]	70.96 ± 8.83	72.38 ± 11.41	71.70 ± 8.84	71.83 ± 9.78
MSRN-MTL [31]	72.29 ± 7.56	73.35 ± 6.24	70.96 ± 7.58	72.85 ± 6.82
Anes-Meta [32]	74.04 ± 9.60	74.91 ± 8.86	75.64 ± 7.37	74.93 ± 8.57
ML-WGCNet (Ours)	76.03 ± 7.46	79.94 ± 7.44	75.77 ± 7.27	78.24 ± 7.34

**Table 6:** Comparison results of ML-WGCNet with the state-of-the-art cross-subject EEG classification methods under different bitter concentrations.

Methods	Accuracy (%)	Precision (%)	Recall (%)	F1-Score (%)
MTL [30]	60.64 ± 10.31	66.91 ± 11.68	61.34 ± 10.39	63.78 ± 10.51
DDA [21]	65.31 ± 10.65	67.17 ± 9.57	66.46 ± 8.25	66.92 ± 6.47
EPEL [23]	65.18 ± 9.98	70.48 ± 10.22	65.40 ± 10.10	68.62 ± 10.27
WBT [22]	72.29 ± 7.66	72.94 ± 9.11	72.91 ± 9.36	72.81 ± 8.35
MC [29]	70.88 ± 8.30	74.21 ± 8.14	71.87 ± 8.18	72.84 ± 6.79
CDSCN [27]	73.81 ± 7.02	74.52 ± 6.14	74.09 ± 7.14	74.10 ± 8.24
MSRN-MTL [31]	72.14 ± 7.76	73.73 ± 7.80	72.37 ± 7.62	73.03 ± 7.65
Anes-Meta [32]	73.37 ± 8.33	78.47 ± 7.79	73.63 ± 8.64	75.90 ± 7.00
ML-WGCNet (Ours)	77.01 ± 7.22	79.53 ± 8.13	78.51 ± 7.37	78.08 ± 7.44

**Table 7:** Comparison of computational complexity between ML-WGCNet and the state-of-the-art cross-subject EEG methods.

Methods	Parameters (M)	Flops (M)
MTL [30]	0.1153	0.9704
DDA [21]	0.7049	1.4092
EPEL [23]	0.6923	1.2341
WBT [22]	0.9345	3.5711
MC [29]	0.8573	4.3611
CDSCN [27]	2.7756	6.2543
MSRN-MTL [31]	0.1417	3.5014
Anes-Meta [32]	0.2546	12.7614
ML-WGCNet (Ours)	0.0207	0.2686

**Table 8:** Statistical test results for different datasets.

Methods	Sweetness Concentrations			Bitter Concentrations		
	t-Value	*p*-Value	Significance	t-Value	*p*-Value	Significance
MTL [30]	7.41	<0.001	***	9.13	<0.001	***
DDA [21]	5.93	<0.001	***	6.35	<0.001	***
EPEL [23]	4.15	<0.001	***	6.82	<0.001	***
WBT [22]	4.51	<0.001	***	3.27	0.002	**
MC [29]	4.21	<0.001	***	4.08	<0.001	***
CDSCN [27]	3.20	0.002	**	2.36	0.020	*
MSRN-MTL [31]	2.78	0.008	**	3.38	0.001	**
Anes-Meta [32]	2.16	0.020	*	2.39	0.020	*

Note: * indicates significance, ** indicates high significance, and *** indicates extremely high significance.

## Data Availability

Dataset available on request from the authors.

## Supplementary Materials

The following supporting information can be downloaded at: https://www.mdpi.com/article/10.3390/bios16050295/s1, Table S1: Classification results of ML-WGCNet using different wavelet basis functions; Table S2: Between-group statistical test results of mean current density values for all brain region under different experimental conditions.
