# Supplementary material for: EEG Cross-Subject Taste Classification Method: A Meta-Learning Wavelet Graph Convolutional Neural Network Under Sweet and Bitter Stimuli
Source: Biosensors (Basel). 2026 May 19;16(5):295. doi: 10.3390/bios16050295 (PMC13204291; doi:10.3390/bios16050295)
Supplement: Supplementary file 1 [file biosensors-16-00295-s001.zip › biosensors-4280466-supplementary.pdf]

biosensors

MDPI

EEG Cross-Subject Taste Classification Method: A Meta-Learning Wavelet Graph Convolutional Neural Network Under Sweet and Bitter Stimuli

In addition to the Morlet wavelet, other commonly used wavelet basis functions in signal processing include the Haar wavelet, Daubechies wavelet, Symlets wavelet, Coiflets wavelet, biorthogonal wavelet, and the Mexican hat wavelet. Table S1 presents the impact of different wavelet basis functions on the classification performance of ML-WGCNet. Among them, the Haar wavelet is simple to compute but suffers from poor smoothness and low frequency resolution, making it difficult to extract fine features from signals. Daubechies, Symlets, and Coiflets are more focused on signal denoising and reconstruction, and their trade-off between time and frequency resolution is weaker than that of the Morlet wavelet. Although the Mexican hat wavelet is similar to the Morlet wavelet in terms of time-frequency distribution, it is a real-valued wavelet and cannot capture phase information. Biorthogonal wavelet is more suitable for image reconstruction tasks and lack advantages in weak feature extraction.

Table S1. Classification results of ML-WGCNet using different wavelet basis functions.

Basis Function	Sweetness Concentrations				Bitter Concentrations			
	Accuracy (%)	Precision (%)	Recall (%)	F1-score (%)	Accuracy (%)	Precision (%)	Recall (%)	F1-score (%)
Haar	68.42±8.34	69.45±7.93	68.32±8.01	68.55±7.94	69.76±9.07	71.23±8.99	69.92±8.32	70.36±8.67
Daubechies	70.45±7.43	71.34±8.03	70.21±7.52	70.79±7.88	71.54±8.76	72.44±8.43	71.22±8.94	71.92±8.66
Symlets	72.56±6.98	73.45±7.33	72.33±7.77	73.01±7.64	70.93±7.33	71.03±7.23	69.72±7.89	70.98±7.66
Coiflets	71.34±7.02	73.84±8.31	71.23±7.38	72.31±8.02	72.27±7.98	72.73±8.82	70.12±7.92	72.22±8.47
Biorthogonal	67.74±7.93	68.32±7.03	67.92±8.37	68.04±7.86	68.93±8.34	70.21±8.03	68.16±7.53	69.03±8.00
Mexican hat	73.48±7.43	74.59±8.44	73.49±7.23	73.96±7.81	73.64±8.01	74.12±7.84	73.88±7.44	74.08±7.69
Morlet (ours)	76.03±7.46	79.94±7.44	75.77±7.27	78.24±7.34	77.01±7.22	79.53±8.13	78.51±7.37	78.08±7.44

Table S2. Between-group statistical test results of mean current density values for all brain region under different experimental conditions.

Comparison	t-Value	p-Value	Significance	Comparison	t-Value	p-Value	Significance
S0 vs S1	3.07	0.012	*	B0 vs B1	3.23	0.009	**
S0 vs S2	3.41	0.003	**	B0 vs B2	3.50	0.005	**
S0 vs S3	3.82	0.006	**	B0 vs B3	3.94	0.002	**
S0 vs S4	4.26	0.001	**	B0 vs B4	4.38	<0.001	***
S0 vs S5	5.12	<0.001	***	B0 vs B5	4.97	<0.001	***
S1 vs S2	2.75	0.021	*	B1 vs B2	2.89	0.016	*
S1 vs S3	3.18	0.004	**	B1 vs B3	3.32	0.007	**
S1 vs S4	3.64	0.009	**	B1 vs B4	3.76	0.003	**
S1 vs S5	5.34	<0.001	***	B1 vs B5	4.85	<0.001	***
S2 vs S3	2.61	0.026	*	B2 vs B3	2.73	0.020	*
S2 vs S4	3.04	0.013	*	B2 vs B4	3.17	0.009	**
S2 vs S5	4.28	<0.001	***	B2 vs B5	4.11	<0.001	***
S3 vs S4	2.52	0.031	*	B3 vs B4	2.64	0.024	*
S3 vs S5	3.84	0.003	**	B3 vs B5	3.82	0.003	**
S4 vs S5	3.14	0.010	*	B4 vs B5	2.93	0.017	*

Note: * indicates significance, ** indicates high significance, and *** indicates extremely high significance.
